# Syntactic and Morphosyntactic Processing in Stroke-Induced and Primary Progressive Aphasia

**DOI:** 10.3233/BEN-2012-110220

**Published:** 2013

**Authors:** Cynthia K. Thompson, Aya Meltzer-Asscher, Soojin Cho, Jiyeon Lee, Christina Wieneke, Sandra Weintraub, M.-Marsel Mesulam

**Affiliations:** ^1^Department of Communication Sciences and DisordersNorthwestern UniversityEvanstonILUSA; ^2^Department of NeurologyNorthwestern UniversityEvanstonILUSA; ^3^Cognitive Neurology and Alzheimer's Disease CenterNorthwestern UniversityEvanstonILUSA; ^4^Department of Psychiatry and Behavioral SciencesNorthwestern UniversityEvanstonILUSA

**Keywords:** Aphasia, primary progressive aphasia, agrammatism, syntactic processing, narrative speech

## Abstract

The paper reports findings derived from three experiments examining syntactic and morphosyntactic processing in individuals with agrammatic and logopenic variants of primary progressive aphasia (PPA-G and PPA-L, respectively) and stroke-induced agrammatic and anomic aphasia (StrAg and StrAn, respectively). We examined comprehension and production of canonical and noncanonical sentence structures and production of tensed and nontensed verb forms using constrained tasks in experiments 1 and 2, using the Northwestern Assessment of Verbs and Sentences (NAVS [57]) and the Northwestern Assessment of Verb Inflection (NAVI, Thompson and Lee, experimental version) test batteries, respectively. Experiment 3 examined free narrative samples, focusing on syntactic and morphosyntactic measures, i.e. production of grammatical sentences, noun to verb ratio, open-class to closed-class word production ratio, and the production of correctly inflected verbs. Results indicate that the two agrammatic groups (i.e., PPA-G and StrAg) pattern alike on syntactic and morphosyntactic measures, showing more impaired noncanonical compared to canonical sentence comprehension and production and greater difficulties producing tensed compared to nontensed verb forms. Their spontaneous speech also contained significantly fewer grammatical sentences and correctly inflected verbs, and they produced a greater proportion of nouns compared to verbs, than healthy speakers. In contrast, PPA-L and StrAn individuals did not display these deficits, and performed significantly better than the agrammatic groups on these measures. The findings suggest that agrammatism, whether induced by degenerative disease or stroke, is associated with characteristic deficits in syntactic and morphosyntactic processing. We therefore recommend that linguistically sophisticated tests and narrative analysis procedures be used to systematically evaluate the linguistic ability of individuals with PPA, contributing to our understanding of the language impairments of different PPA variants.

## 1. Introduction

A large body of literature exists, which describes the linguistic deficits associated with different types of aphasia caused by stroke (StrAph). Many individuals with stroke-induced agrammatic aphasia (StrAg) of Broca's type present a characteristic pattern of nonfluent speech, as well as grammatical, or (morpho)syntactic, deficits. The latter are exhibited by impaired comprehension and production of sentences with complex syntactic structures, in particular semantically reversible noncanonical sentences involving syntactic movement (e.g. [9,11,31,62]), impaired production of grammatical morphemes, in particular tense inflec-

ISSN 0953-4180/13/$27.50 © 2013 – IOS Press and the authors. All rights reserved tion (e.g. [1,22,58]), and greater difficulty producing verbs as compared to nouns (e.g. [44,69]). In contrast, stroke-induced anomic aphasic (StrAn) individuals speak fluently and present with intact syntactic abilities, but evince significant naming difficulties, primarily for nouns [24–26,44,69].

Language deficits have also been reported for individuals with primary progressive aphasia (PPA), a language impairment caused by a neurodegenerative disease [39,40]. According to current guidelines, PPA can be subdivided into three variants: agrammatic/nonfluent (PPA-G), logopenic (PPA-L), and semantic (PPA-S) [27,29,42]. Individuals with the PPA-G variant present with nonfluent speech, verb production deficits, and difficulties with comprehension and production of syntactically complex sentences [13,32,34, 48,55,64,67]. Conversely, PPA-L is associated with fluctuating fluency, difficulties with word retrieval, and impaired repetition, with relatively spared syntax and word comprehension [28], and individuals with PPA-S show relatively fluent patterns of speech production, but have problems with naming and comprehension of single words [35,41,42]. The term progressive nonfluent aphasia (PNFA) used in the earlier literature has inconsistently included what is now known as PPA-G and PPA-L patients without making a distinction between the two groups.

Importantly, the underlying neurological deficits of the PPA and StrAph clinical populations are distinctly different. Areas of the brain impaired by stroke follow the territory of the cerebral vascular system, whereas those associated with PPA do not. In addition, neurodegenerative disease, as in PPA, affects only particular groups and layers of cells, leaving others relatively intact. Hence, affected regions of the brain can remain active during language task performance [52]. Nevertheless, in general, PPA-G and StrAg are associated with necrosed or atrophied tissue in the left frontal region, although tissue atrophy in PPA-G also may involve the temporo-parietal junction (TPJ) and lesions in StrAg can include both anterior and posterior perisylvian brain regions [44,56,65]. Conversely, PPA-L and StrAn typically result from compromised tissue in the temporo-parietal region. PPA-L is correlated with atrophy in the left TPJ, and the adjacent parts of the lateral temporal cortex [27,42] and classic StrAn often results from damage to posterior temporal and parietal regions, which tends to spare the superior temporal gyrus [36], although some anomic aphasic individuals present with left anterior temporal lobe lesions, including the temporal pole and inferior temporal gyrus [15]. Finally, PPA-S results from anterior temporal atrophy, more pronounced in the left hemisphere [27,42].

Despite these neuropathophysiological differences between PPA and StrAph there is some evidence, at least for the two agrammatic groups, i.e., PPA-G and StrAg, that they present with similar behavioral deficits. For example, Thompson et al. [60] examined production and comprehension of nouns and verbs in PPA and StrAph individuals, using the Northwestern Naming Battery [63] and found similar behavioral patterns for PPA-G and StrAg participants. Both groups had greater difficulty naming verbs compared to nouns, and showed effects of argument structure complexity on verb production. However, it is still unclear whether the grammatical impairment in PPA-G is parallel to that in StrAg. For example, Graham et al. [30] argue that PNFA is not characterized by agrammatism, as the speech of individuals with this PPA subtype includes normal proportions of content and function words as well as nouns and verbs, unlike that of StrAg speakers. This conclusion is difficult to interpret since their PNFA group may have included a mixture of PPA-G and PPA-L. Furthermore, to our knowledge, syntactic and morphosyntactic impairments associated with PPA and StrAph have not been examined systematically and no studies have compared language deficit patterns in other types of stroke-induced aphasia (e.g., StrAn) with those found in other PPA variants (e.g., PPA-L).

The aim of the present study is to explore (morpho)syntactic impairment patterns in individuals with PPA-G and PPA-L and to compare them with individuals with StrAg and StrAn. We examined comprehension and production of canonical and noncanonical sentences, production of verb inflection and other syntactic and morphosyntactic variables in three experiments. As detailed below, in Experiments 1 and 2 constrained tasks were used to evaluate sentence comprehension and production abilities as well as production of grammatical morphemes, i.e., the Northwestern Assessment of Verbs and Sentences (NAVS [54]) and the Northwestern Assessment of Verb Inflection (NAVI; Lee and Thompson, experimental version), respectively. Experiment 3 included analysis of free narrative speech samples obtained from participants.

## 2. Experiment 1. Patterns of sentence comprehension and production

Whereas canonical sentences in English display the subject-verb-object order typical of this language (e.g.

Table 1
Demographic data for paticipants in Experiments 1, 2, and 3

| Participants | N | Males | Handedness (left/right) | Age | Education | Duration of symptoms (years) |
|---|---|---|---|---|---|---|
| *Experiment 1* | | | | | | |
| Primary Progressive Aphasia (PPA) | | | | | | |
| PPA-G | 11 | 6 | 1/10 | 63.8 | 16.3 | 3.3 |
| PPA-L | 15 | 9 | 2/13 | 62.9 | 16.1 | 3.9 |
| Stroke Aphasia (StrAph) | | | | | | |
| StrAg | 22 | 16 | 2/20 | 53.7 | 16.7 | 5.8 |
| StrAn | 18 | 10 | 4/14 | 59.8 | 15.6 | 4.9 |
| *Experiment 2* | | | | | | |
| Primary Progressive Aphasia (PPA) | | | | | | |
| PPA-G | 7 | 2 | 0/7 | 64.1 | 16.0 | 2.7 |
| PPA-L | 10 | 6 | 0/10 | 66.2 | 16.9 | 2.8 |
| Stroke Aphasia (StrAph) | | | | | | |
| StrAg | 20 | 14 | 2/18 | 55.3 | 16.7 | 6.5 |
| StrAn | 9 | 5 | 2/7 | 56.2 | 15.4 | 3.0 |
| *Experiment 3* | | | | | | |
| Primary Progressive Aphasia (PPA) | | | | | | |
| PPA-G | 9 | 5 | 1/8 | 63.0 | 16.6 | 3.8 |
| PPA-L | 15 | 9 | 2/13 | 66.5 | 15.9 | 3.7 |
| Stroke Aphasia (StrAph) | | | | | | |
| StrAg | 8 | 5 | 1/7 | 50.3 | 16.3 | 4.6 |

'The dog chased the cat'), in noncanonical structures the object is moved across the verb and the subject, and surfaces in a clause-initial position (e.g. 'The cat was chased by the dog') (see e.g. [12]). Many studies have established that individuals with StrAg have difficulties comprehending and producing noncanonical sentences that are semantically reversible [9,11,31,62]. This pattern also has been noted in PPA-G in production, using the Northwestern Anagram Test (NAT [63, 67]). However, patterns of canonical and noncanonical sentence comprehension have not been established in PPA-G or other PPA variants and the performance of PPA and StrAph groups have not been directly compared with one another. In the present experiment we used the Sentence Comprehension Test (SCT) and Sentence Production Priming Test (SPPT) from the NAVS to compare comprehension and production of canonical and noncanonical sentences in participant groups with PPA and StrAph.

### 2.1. Method

#### 2.1.1. Participants

Eleven PPA-G, 15 PPA-L, 22 StrAg and 18 StrAn volunteers, recruited from the subject pools of the Aphasia and Neurolinguistics Research Laboratory in Evanston, IL and the Cognitive Neurology and Alzheimer's Disease Center in Chicago, IL, participated in the study. All were native, monolingual English speakers, with normal or corrected-to-normal hearing and vision. Three of the PPA participants and six of the StrAph ones were left handed, and the rest were right handed. The four groups were matched for education (Kruskal Wallis, $\chi^2(3) = 2.84$, $p = 0.417$). Participants ranged in age from 35 to 79 years (mean 63.8 yrs for PPA-G; 62.9 yrs for PPA-L; 53.6 yrs for StrAg; 59.8 yrs for StrAn), with the PPA participants, in general, older than the StrAph participants (Mann-Whitney, $Z = -2.61, p = 0.009$). The four groups were, therefore, not completely matched for age (Kruskal Wallis, $\chi^2(3) = 9.9$, $p = 0.019$), although there was no significant difference between the PPA-G and PPA-L groups (Mann-Whitney, $Z = -1.56$, $p = 0.876$) or between the StrAg and StrAn groups (Mann-Whitney, $Z = -1.6, p = 0.102$) (see Table 1). All participants provided informed consent, and the study was approved by the Institutional Review Board at Northwestern University.

The diagnosis of PPA was based on neurological examination, clinical presentation, and neuropsychological test performance. None of the PPA patients showed evidence of stroke or other neurological disorder and all presented a history of progressive language deficits in the face of relatively spared performance in other cognitive domains. To rule out memory and attention deficits we administered the Mini-Mental State Examination (MMSE) [21], Wechsler Memory Scale (WMS) [66], Facial Recognition [7], and Trail Making Tests [46] (see test scores in Table 2). Twenty-two of the 26 PPA participants achieved a score of 24 or higher on the MMSE, indicating normal performance. The remaining four achieved scores between 16 and 23, suggesting mild impairment, (but importantly, scores on this measure are influenced by patients' compromised language ability [23,45]).

PPA variants were determined based on single word comprehension ability, measured by the Peabody Picture Vocabulary Test (PPVT [18]) (moderately difficult items, #157–192), and on canonical and noncanonical sentence generation, measured by the NAT (see scores in Table 3). Individuals showing relatively spared single word comprehension as well as sentence generation (a score of 60% or higher on both the PPVT and the NAT) were classified as PPA-L, whereas speakers showing spared single word comprehension (60% or higher on the PPVT) but impaired sentence generation (a score lower than 60% on the NAT) were classified as PPA-G. Accordingly, both the PPA-G and the PPA-L groups showed relatively spared word comprehension (PPA-G PPVT mean: 95.2; PPA-L PPVT mean: 92.9), with no significant difference between the groups (Mann-Whitney, $Z = -0.447$, $p = 0.655$). On the NAT, the two groups performed in a similar manner on canonical sentences (Mann-Whitney, $Z = -1.27$, $p = 0.204$), but differed significantly on noncanonical sentences (Mann-Whitney, $Z = -3.43$, $p = 0.001$), with PPA-G participants showing poorer performance, consistent with their syntactic impairment.

To further assess the linguistic impairment of the PPA participants, the Western Aphasia Battery (WAB [36]) was administered, and Aphasia Quotients (AQs) were computed based on WAB subtest scores (see Table 3). Mean WAB-AQ for the PPA-G participants was 82.95 (range: 75.3–95.2), and for the PPA-L participants was 88.5 (range: 74.4–95.8). WAB-AQs differed significantly between the two groups (Mann-Whitney $Z = -2.18$, $p = 0.027$), reflecting significant differences between the two groups on WAB fluency scores (Mann-Whitney, $Z = -3.01$, $p = 0.002$). However, the groups did not differ significantly on the auditory comprehension (Mann-Whitney, $Z = -1.2$, $p = 0.237$), repetition[1] (Mann-Whitney, $Z = -1.49$, $p = 0.148$) or naming (Mann-Whitney, $Z = -0.47$, $p = 0.646$) subtests of the WAB.

All of the StrAph participants suffered left-hemisphere strokes, on average 5.4 years prior to the study. Aphasia type was determined by participants' performance on the WAB (also shown in Table 3) as well as clinical impression. Participants were classified as agrammatic if their spontaneous speech was nonfluent, consisting of short, simple phrases with omission of some grammatical morphemes. Conversely, participants were diagnosed as anomic if their speech was fluent, with primarily word retrieval difficulties. Accordingly, the StrAg participants in the experiment presented with mild-to-moderate aphasia (WAB-AQ mean: 77.13, range: 66–87.2) and nonfluent speech (WAB fluency mean: 4.5, range 4–5). The StrAn participants also presented with mild-to-moderate aphasia (WAB-AQ mean: 85.6, range: 69.4–92.4), but with fluent speech (WAB fluency mean: 7.6, range: 6–9) and mild-to-moderate word retrieval difficulty. WAB-AQs for the two groups differed significantly (Mann-Whitney, $Z = -3.78$, $p < 0.001$) largely because of significant differences in fluency scores (Mann-Whitney $Z = -5.08$, $p < 0.001$). The two groups also differed on the WAB repetition test (Mann-Whitney $Z = -2.26$, $p = 0.024$), but not on the auditory comprehension (Mann-Whitney $Z = -1.89$, $p = 0.058$) or naming subtests (Mann-Whitney $Z = -0.613$, $p = 0.545$).

Comparing the PPA and StrAph groups' WAB-AQ scores, there were no significant differences between the PPA-L and StrAn groups (Mann-Whitney, $Z = 1.57$, $p = 0.11$), but a significant difference was found between the PPA-G and StrAg groups (Mann-Whitney, $Z = 2.54$, $p = 0.011$), stemming from a significant difference in naming subtest scores (Mann-Whitney, $Z = 2.21$, $p = 0.027$), such that the PPA-G participants performed better on this subtest than the StrAg participants. However, there were no significant differences between the two agrammatic groups on other subtests of the WAB.

### 2.1.2. Materials and procedure

Participants were tested using the SCT and SPPT subtests from the NAVS, with the former always administered prior to the latter. The SCT and SPPT items included 30 sentences, five sentences for each of six sentence types: actives, subject wh-questions, subject relative clauses, passives, object wh-questions, and object relative clauses. The first three constructions are canonical, whereas, the latter three are noncanonical. All sentences were semantically reversible with world-knowledge alone insufficient for determining which participant was the agent (doer of the action) and which the theme (recipient of the action). Table 4 contains examples of the sentence types tested. Picture stim-

[1]Gorno-Tempini et al. [29] proposed a classification system, according to which PPA-L individuals should present with impaired repetition of sentences and phrases, consistent with the notion that these individuals have a phonological short-term memory deficit. The subtyping criteria used here are those suggested by Mesulam et al. [42], which do not include repetition.

Table 2
Mean neuropsychological scores (standard deviations) for PPA participants in Experiments 1, 2, and 3

| Participants | MMSE (30) | WMS-iii | | Visual perception | Processing speed |
|---|---|---|---|---|---|
| | | Immediate (48) | Delayed (48) | Facial Rec (54) | Trail making test A |
| Experiment 1 | | | | | |
| PPA-G | 27.2 (2.82) | 34.0 (4.24) | 35.0 (4.52) | 47.9 (3.21) | 58.4 (35.26) |
| PPA-L | 25.7 (4.11) | 35.8 (5.21) | 38.9 (5.45) | 46.6 (3.74) | 44.6 (21.73) |
| Experiment 2 | | | | | |
| PPA-G | 27.8 (1.95) | 34.0 (4.08) | 34.1 (4.3) | 46.9 (3.39) | 59.3 (38.43) |
| PPA-L | 28.0 (1.63) | 33.6 (4.12) | 35.8 (4.13) | 48.8 (3.12) | 32.2 (9.59) |
| Experiment 3 | | | | | |
| PPA-G | 27.2 (2.99) | 34.4 (4.53) | 36.4 (3.7) | 48.9 (3.62) | 48.3 (25.6) |
| PPA-L | 25.1 (5.57) | 34.6 (4.79) | 38.7 (5.46) | 47.5 (3.82) | 53.2 (29.99) |

Note: MMSE = Mini-Mental State Exam, WMS = Wechsler Memory Scale, Facial Rec. = Facial Recognition.

uli used for the SCT and the SPPT depicted reversible scenes corresponding with each sentence, such as a cat watching a dog and a dog watching a cat (see Fig. 1).

In the SCT, participants were presented with a randomly selected picture pair while a target sentence, corresponding to one of the pictures, was read aloud by the examiner. Participants were then asked to point to the picture depicting the scenario described. A 10-sec response time was allowed, and if no response occurred within the allotted time, or if requested, sentences were repeated once. Responses were counted as correct if provided within the given time, and if the chosen picture matched the target sentence.

In the SPPT, for each picture pair presented, the examiner modeled the target sentence structure, describing the picture on the participant's left. The participant was then asked to produce a sentence 'just like it' for the picture on the right. Target sentence types were tested in blocks, with five exemplars of each type presented consecutively. A 15-sec response time was provided for each trial, which was repeated once if no response or an incorrect response was produced. Grammatical sentences of the target syntactic structure produced within the allotted time were counted as correct, with any inflection or paraphasic (either phonological or semantic) errors accepted, unless phonological errors resulted in nonwords, or semantic errors reversed the agent and theme of target sentences.

### 2.2. *Results*

Percent correct production (SPPT) and comprehension (SCT) of each sentence type for the PPA and StrAph participant groups are presented in Tables 5 and 6, respectively and in Fig. 2. A 2 (group) x 2 (modality) x 2 (canonicity) ANOVA for the PPA participants revealed significant main effects of group ($F$ (1, 23) = 5.87, $p$ = 0.024), with PPA-G showing poorer performance than PPA-L, modality ($F$ (1, 23) = 9.55, $p$ = 0.005), with production more difficult than comprehension, and sentence canonicity ($F$ (1, 23) = 39.61, $p < 0.001$), with noncanonical sentences more difficult than canonical ones. A significant two-way interaction effect also was found between modality and canonicity ($F$ (1, 23) = 8.13, $p$ = 0.009), indicating that the difference between canonical and noncanonical sentences was greater for production compared to comprehension. Additionally, there was a significant interaction between group and canonicity ($F$ (1, 23) = 10.73, $p$ = 0.003), with the canonicity effect greater for the PPA-G than for the PPA-L group. There was also a significant group x canonicity x modality interaction ($F$ (1, 23) = 9.328, $p$ = 0.006). Post-hoc analysis using the Mann-Whitney test revealed that the PPA-L group performed significantly better than the PPA-G group in production of noncanonical sentences ($Z = -2.19$, $p$ = 0.028). Further, the difference between groups for comprehension of noncanonical sentences approached significance ($Z = -1.72$, $p$ = 0.085). However, the two groups did not differ in production or comprehension of canonical forms ($Z = -.0784$, $p$ = 0.433 and $Z = -1.28$, $p$ = 0.281, respectively).

For the StrAph data, a 2 × 2 × 2 ANOVA revealed a main effect of group ($F$ (1, 35) = 5.75, $p$ = 0.021), with StrAg showing poorer performance than StrAn, as well as a main effect of modality ($F$ (1, 35) = 14.01, $p$ = 0.001) and canonicity ($F$ (1, 35) = 54.28, $p <$ 0.001) similar to those found in the PPA analysis. Significant interactions were also found between modality and canonicity ($F$ (1, 35) = 10.99, $p$ = 0.002), and between canonicity and group ($F$ (1, 35) = 5.4, $p$ = 0.026). Although we did not find a three-way group x canonicity x modality interaction, follow-up comparisons between the StrAg and StrAn groups, us-

Table 3
Mean language scores (standard deviations) for PPA and stroke-induced aphasic groups in Experiments 1, 2, and 3

| Participants | Western aphasia battery | | | | | PPVT | NAT | |
|---|---|---|---|---|---|---|---|---|
| | AQ (100) | F (10) | Comp (10) | Rep (10) | Nam (10) | (%) | C (%) | NC (%) |
| *Experiment 1* | | | | | | | | |
| Primary Progressive Aphasia (PPA) | | | | | | | | |
| PPA-G | 82.9 (5.8) | 5.6 (1.7) | 9.3 (0.6) | 8.0 (1.0) | 9.1 (0.4) | 95.2 (4.3) | 86.6 (17.0) | 41.5 (14.1) |
| PPA-L | 88.5 (6.1) | 8.0 (1.3) | 9.5 (0.6) | 8.6 (1.0) | 8.7 (1.5) | 92.9 (8.3) | 95.6 (6.5) | 78.3 (18.2) |
| Stroke Aphasia (StrAph) | | | | | | | | |
| StrAg | 77.1 (5.8) | 4.5 (0.5) | 8.8 (1.0) | 8.1 (1.2) | 8.4 (1.1) | | | |
| StrAn | 85.6 (6.4) | 7.6 (1.6) | 9.4 (0.6) | 9.0 (0.8) | 8.1 (1.3) | | | |
| *Experiment 2* | | | | | | | | |
| Primary Progressive Aphasia (PPA) | | | | | | | | |
| PPA-G | 84.2 (7.1) | 6.0 (1.9) | 9.4 (0.7) | 8.5 (0.8) | 9.1 (0.3) | 95.2 (4.5) | 85.7 (18.6) | 43.8 (15.3) |
| PPA-L | 91.5 (4.7) | 8.5 (1.4) | 9.7 (0.5) | 9.1 (0.9) | 8.7 (1.5) | 92.8 (8.6) | 95.3 (6.30) | 90 (12.3) |
| Stroke Aphasia (StrAph) | | | | | | | | |
| StrAg | 78.3 (6.7) | 4.5 (0.5) | 9.1 (0.9) | 8.0 (1.3) | 8.5 (0.9) | | | |
| StrAn | 87.4 (7.5) | 8.0 (1.3) | 9.5 (0.7) | 8.7 (0.8) | 8.5 (1.9) | | | |
| *Experiment 3* | | | | | | | | |
| Primary Progressive Aphasia (PPA) | | | | | | | | |
| PPA-G | 79.4 (3.4) | 5.0 (1.0) | 9.0 (0.7) | 7.4 (1.2) | 9.1 (0.5) | 94.8 (4.3) | 76.2 (26.3) | 30.5 (18.0) |
| PPA-L | 88.4 (5.4) | 8.1 (1.2) | 9.6 (0.5) | 8.5 (1.0) | 8.6 (1.5) | 94.0 (7.8) | 94.5 (6.5) | 78.2 (16.4) |
| Stroke Aphasia (StrAph) | | | | | | | | |
| StrAg | 75.3 (6.9) | 4.4 (0.7) | 8.6 (1.0) | 7.7 (1.0) | 8.0 (1.6) | | | |

*Note*: AQ = Aphasia Quotient, F = Fluency, Comp = Auditory Comprehension, Rep = Repetition, Nam = Naming, PPVT = Peabody Picture Vocabulary Test, NAT = Northwestern Anagram Test, C = Canonical, NC = Noncanonical.

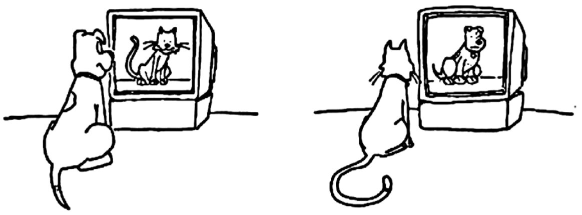

Fig. 3. Example of a picture stimulus pair used in the NAVS-SCT and NAVS-SPPT in Experiment 1.

ing the Mann-Whitney test, were performed. These analyses revealed that the StrAg group's performance was significantly poorer than that of the StrAn group for production of noncanonical sentences ($Z = -2.78$, $p = 0.005$) and the difference in comprehension of noncanonical sentences approached significance ($Z = -1.91$, $p = 0.056$). However, the two groups did not differ significantly in the comprehension ($Z = -1.53$, $p = 0.125$) or production ($Z = -1.1$, $p = 0.272$) of canonical sentences.

Statistical comparison between the two agrammatic groups, namely PPA-G and StrAg, yielded no main effect of aphasia type ($F$ (1, 31) = 0.078, $p = 0.782$) (see Fig. 2). There was a significant effect of modality ($F$ (1, 31) = 16.03, $p < 0.001$), with production more impaired than comprehension, and of canonicity ($F$ (1, 31) = 49.85, $p < 0.001$), with noncanonical sentences more difficult than canonical ones. There was also a significant two-way interaction between modality and canonicity ($F$ (1, 31) = 13.35, $p = 0.001$), with the canonicity effect larger for production than for comprehension.

### 2.3. Discussion

This experiment revealed considerable similarities between PPA and StrAph. In both groups, individuals with agrammatic deficits (namely, PPA-G and StrAg) were significantly more impaired in production of noncanonical sentences than participants with the logopenic variant of PPA or stroke-induced anomic aphasia. Likewise, there was a trend toward impaired comprehension of noncanonical sentences in the two agrammatic groups, compared to the other two groups. Interestingly, the deficits did not extend to canonical sentences, on which production and comprehension for the agrammatic participants was comparable to that of the logopenic and anomic groups. The selective deficit in noncanonical sentences for agrammatic speakers indicates a syntactically related impairment. It is important to point out that deficits in comprehension of noncanonical sentence comprehension may be related to general impairments in verbal working memory (e.g., [10]; but see [51] for arguments against this view). However, we find that a working memory account does not completely explain the deficit patterns

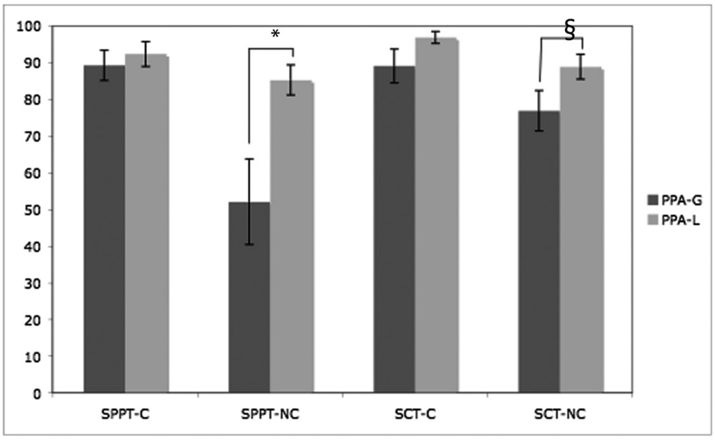

(a)

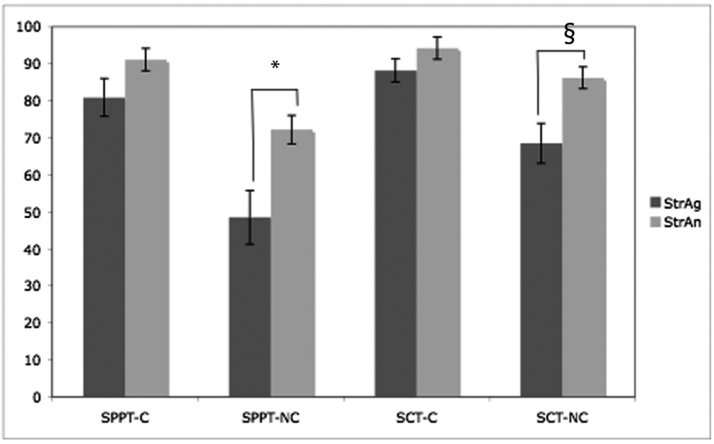

(b)

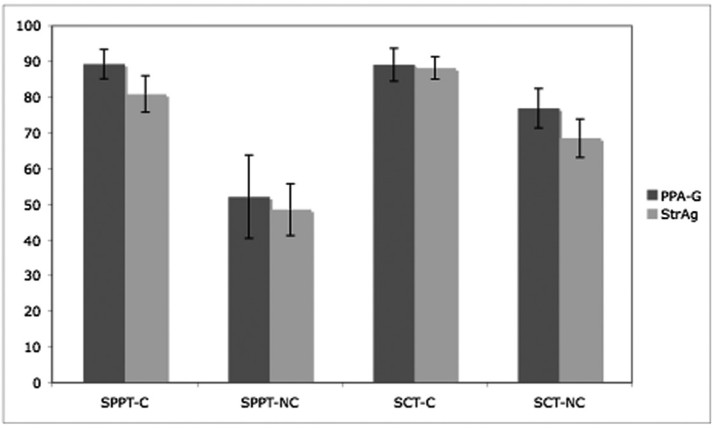

(c)

Fig. 2. Percent correct responses (and standard error), Experiment 1: NAVS Sentence Production Priming Test (SPPT) and Sentence Comprehension Test (SCT) for (a) PPA and (b) StrAph participants for canonical (C) and noncanonical sentences (NC) sentences; (c) depicts results for the two agrammatic groups. §$p < 0.1$; $^{*}p < 0.05$, Mann-Whitney test, two-tailed.

Table 4

| Sentence class | Sentence type | Example |
|---|---|---|
| Canonical | Active | The dog was watching the cat |
| | Subject relative | Pete saw the dog who was watching the cat |
| | Subject Wh-question | Who was watching the cat? |
| Non-canonical | Passive | The cat was watched by the dog |
| | Object relative | Pete saw the cat who the dog was watching |
| | Object Wh-question | Who was the dog watching? |

Table 5
Percent correct responses (standard deviation) for PPA participants in Experiment 1

| Test | Participants | Active | SWQ | SR | Passive | OWQ | OR |
|---|---|---|---|---|---|---|---|
| SPPT | PPA-G | 100 (0.0) | 88.0 (25.3) | 80.0 (35.3) | 54.0 (47.2) | 70.0 (38.0) | 32.0 (42.4) |
| | PPA-L | 97.3 (10.3) | 93.3 (12.3) | 86.7 (27.9) | 92.0 (16.6) | 96.0 (15.5) | 68.0 (32.8) |
| SCT | PPA-G | 94.5 (9.3) | 87.3 (20.5) | 85.4 (23.8) | 70.9 (35.1) | 83.6 (17.5) | 74.5 (20.2) |
| | PPA-L | 97.3 (7.0) | 94.7 (11.9) | 98.7 (5.20) | 93.3 (12.3) | 86.7 (19.5) | 86.7 (16.3) |

*Note*: SPPT = Sentence Production Priming Test, SCT = Sentence Comprehension Test, SWQ = Subject Wh-Questions, OWQ = Object Wh-Questions, SR = Subject Relatives, OR = Object Relatives.

Table 6
Percent correct responses (standard deviation) for StrAph participants in Experiment 1

| Test | Participants | Active | SWQ | SR | Passive | OWQ | OR |
|---|---|---|---|---|---|---|---|
| SPPT | StrAg | 94.5 (17.6) | 74.5 (33.3) | 73.6 (38.2) | 50.9 (44.0) | 65.4 (41.5) | 29.1 (36.4) |
| | StrAn | 97.8 (6.5) | 86.7 (25.7) | 88.9 (18.4) | 85.6 (22.5) | 90.0 (20.9) | 41.0 (38.5) |
| SCT | StrAg | 90.9 (16.0) | 83.6 (24.4) | 90.0 (17.2) | 67.3 (34.7) | 77.7 (21.6) | 60.9 (33.5) |
| | StrAn | 100 (0.0) | 86.7 (28.9) | 96.0 (11.2) | 89.3 (18.3) | 88.0 (22.4) | 81.3 (22.0) |

*Note*: SPPT = Sentence Production Priming Test, SCT = Sentence Comprehension Test, SWQ = Subject Wh-Questions, OWQ = Object Wh-Questions, SR = Subject Relatives, OR = Object Relatives.

found here. Notably, comprehension of subject-relative constructions, which are canonical and similar in length to object relative constructions, was superior to object-relative structures (85.4% correct for subject relatives, 74.5% correct for object relatives for PPA-G; 90% for subject relatives compared to 60.9% for object relatives for StrAg).

Another interesting result of the experiment is that the PPA-G and the StrAg groups performed very similarly, with no significant differences found for any tasks or condition. This indicates that PPA-G individuals indeed exhibit noncanonical sentence deficits in line with those seen in stroke-induced agrammatic speakers.

## 3. Experiment 2. Production of verb inflections

A large number of studies have shown that StrAg aphasic individuals have difficulty with inflectional morphology, shown by a tendency to omit or substitute grammatical morphemes that mark tense, aspect and agreement [1,4,22,37,58]. In particular, finite verb forms, inflected for tense (e.g., the past-tense form '*fixed*'), are especially vulnerable to disruption in stroke-induced agrammatism, whereas nonfinite verb forms (e.g. the infinitive '*to fix*') are generally spared [5,17,19,20]; also see Thompson et al. [59] for review. However, to our knowledge no studies have systematically investigated grammatical morphology deficits related to tense in individuals with PPA. Because difficulty producing grammatical morphemes, particularly those marking verb tense, is one of the major deficits seen in StrAg, investigation of inflectional morphology is important for detailing morphosyntactic deficits in PPA-G. Further, detailing patterns of impaired/spared grammatical morphology across PPA subtypes will help to distinguish between them. In this experiment, we therefore compared PPA and StrAph participant groups on verb inflection production, using the NAVI, a test designed to evaluate production of nonfinite and finite verb forms.

### 3.1. Participants

Participants included 7 PPA-G, 10 PPA-L, 20 StrAg and 9 StrAn individuals, recruited from the subject pools of the Aphasia and Neurolinguistics Research Laboratory in Evanston, IL and the Cognitive Neurology and Alzheimer's Disease Center in Chicago, IL. The participants met the same selection criteria as in

Experiment 1. All 17 PPA participants, and 25 of the 29 StrAph participants, were right handed. The groups were matched for education (Kruskal Wallis, $\chi^2(3) = 3.36$, $p = 0.339$) and age (Kruskal-Wallis, $\chi^2(3) = 7.64$, $p = 0.054$) (see Table 1).

The PPA participants recruited for the study showed no evidence of stroke or other neurological disorder and all presented a history of progressive language deficits with relatively spared performance in other cognitive domains. As in Experiment 1, the MMSE, WMS, Facial Recognition and Trail Making Tests were administered to rule out possible memory, visual perceptual, and attention deficits (see scores in Table 2). All participants scored 24 or higher on the MMSE.

As in Experiment 1, PPA variants were determined based on scores in the PPVT and the NAT. Both the PPA-G and the PPA-L groups showed relatively spared word comprehension, with no significant difference between groups (PPA-G PPVT mean: 95.2; PPA-L PPVT mean: 92.8, Mann-Whitney, $Z = -0.453$, $p = 0.650$). Further, on the NAT, the two groups did not differ significantly on canonical sentences (PPA-G mean: 85.7, PPA-L mean: 95.3, Mann-Whitney, $Z = -0.959$, $p = 0.338$). However, the PPA-G group was significantly more impaired than the PPA-L group on noncanonical sentences (PPA-G mean: 43.8, PPA-L mean: 90.0, Mann-Whitney, $Z = -3.45$, $p = 0.001$).

We also administered the WAB to all PPA participants, with mean AQs of 84.2 (range: 75.3–95.2) and 91.5 (range: 83.2–97.2), for the PPA-G and PPA-L participants, respectively, which again differed significantly between groups (Mann-Whitney, $Z = -2.0$, $p = 0.045$) due to significant differences in WAB fluency scores (Mann-Whitney, $Z = -2.39$, $p = 0.017$). However, the groups did not differ significantly on the auditory comprehension (Mann-Whitney, $Z = -0.86$, $p = 0.389$), repetition (Mann-Whitney, $Z = -1.468$, $p = 0.142$) or naming (Mann-Whitney, $Z = -0.245$, $p = 0.806$) subtests of the WAB.

The stroke aphasic participants all suffered a left hemisphere stroke, with the exception of one, who presented with a right hemisphere lesion and crossed aphasia, with an average of 5.6 years post onset. Aphasia type was determined as in Experiment 1. All participants presented with mild-to-moderate aphasia (WAB-AQ mean: StrAg: 78.3, range 65.5–87.6; StrAn: 87.4, range: 69.4–93.3), with WAB fluency scores ranging from 4 to 5 (mean = 4.5) for the former group and from 6 to 9 (mean = 8.0) for the latter. As in Experiment 1, statistical analyses comparing the two groups indicated significant differences in WAB-AQ (Mann-Whitney, $Z = -3.39$, $p = 0.001$) and fluency scores (Mann-Whitney, $Z = -4.44$, $p < 0.001$), but the two groups did not differ significantly on the auditory comprehension (Mann-Whitney, $Z = -0.33$, $p = 0.74$), repetition (Mann-Whitney, $Z = -1.53$, $p = 0.125$), or naming (Mann-Whitney, $Z = -1.56$, $p = 0.119$) subtests of the WAB. (See Table 3 for language test scores for the PPA and StrAph groups.)

The two agrammatic groups, PPA-G and StrAg, did not differ on their WAB-AQs (Mann-Whitney, $Z = 0.1772$, $p = 0.081$). Similarly, there was no difference in WAB-AQ between the PPA-L and the StrAn group (Mann-Whitney, $Z = 1.103$, $p = 0.278$).

### 3.2. *Materials and procedure*

Ten two-argument (transitive) verbs were selected, including five with regular (e.g., *tickle*) and five with irregular (e.g., *eat*) past-tense morphology, with each elicited in five different forms: infinitive (e.g., *to eat*), progressive (e.g., *is eating*), present singular (e.g., *eats*), present plural (e.g., *eat*), past regular (for regular verbs, e.g., *tickled*), and past irregular (for irregular verbs, e.g., *ate*), resulting in a total of 50 experimental trials (see Table 7 for a set of sample stimuli). Note that the first two verb forms (i.e., the infinitive and progressive) are nonfinite – that is, uninflected for tense (in the progressive, tense is marked on the auxiliary verb, and the lexical verb is a participle), and the latter four are finite forms – inflected for tense. Black-and-white line drawings were prepared for all verbs, depicting an agent and a theme (e.g., a man eating a hamburger) to elicit all verb forms, except for the present plural, for which two agents and two themes were illustrated (e.g., two men eating hamburgers).

A sentence completion task, using temporal adverbs such as *everyday* (to elicit present tense singular and plural forms) and *yesterday* (to elicit past tense forms), was used to obligate production of each target verb form. Together with each stimulus picture, a sentence with a missing verb was presented and read aloud and participants were asked to complete the sentence by providing the correct verb form. To control for verb retrieval difficulties, the verb stem of each target form also was provided on each stimulus template (see Fig. 3). Responses were counted as correct if the target verb form was produced within 10 seconds of stimulus presentation. For any responses with self-corrections, the last attempt was scored.

Table 7
Verb forms assessed by the NAVI and corresponding example sentences used to elicit them in Experiment 2

| Finiteness | Verb form | Example |
|---|---|---|
| Nonfinite (Uninflected for tense) | Infinitive | The man likes to *eat* the hamburger. |
| | Progressive | Now the man is *eating* the hamburger. |
| Finite (Inflected for tense) | Present singular | Everyday the man *eats* the hamburger. |
| | Present plural | Everyday the men *eat* the hamburgers. |
| | Past – regular | Yesterday the boy *tickled* the girl. |
| | Past – irregular | Yesterday the man *ate* the hamburger. |

Table 8
Percent correct responses of StrAph and PPA participants for nonfinite and finite verb forms, Experiment 2

| Participant | Nonfinite verb forms | | | | Finite verb forms | | | |
|---|---|---|---|---|---|---|---|---|
| | Inf. | Prog. | Total | Pres. sin. | Pres. pl. | Past reg. | Past irreg. | Total |
| Primary Progressive Aphasia (PPA) | | | | | | | | |
| PPA-G | 92.4 (14.6) | 96.2 (7.6) | 94.3 (11.0) | 61.0 (40.5) | 59.0 (28.3) | 80.0 (28.3) | 71.4 (25.4) | 66.7 (21.5) |
| PPA-L | 99.0 (3.20) | 99.0 (3.2) | 99.0 (3.20) | 92.3 (8.0) | 75.3 (29.4) | 95.0 (12.7) | 84.0 (24.6) | 87.6 (10.4) |
| Stroke-induced aphasia (StrAph) | | | | | | | | |
| StrAg | 87.3 (26.2) | 90.8 (14.7) | 89.1 (16.7) | 67.8 (35.7) | 41.9 (30.8) | 55.0 (41.0) | 48.0 (38.6) | 53.2 (27.5) |
| StrAn | 100 (0.0) | 97.0 (4.6) | 98.5 (2.3) | 80.3 (12.0) | 65.6 (28.3) | 76.7 (22.4) | 76.7 (22.4) | 74.8 (18.9) |

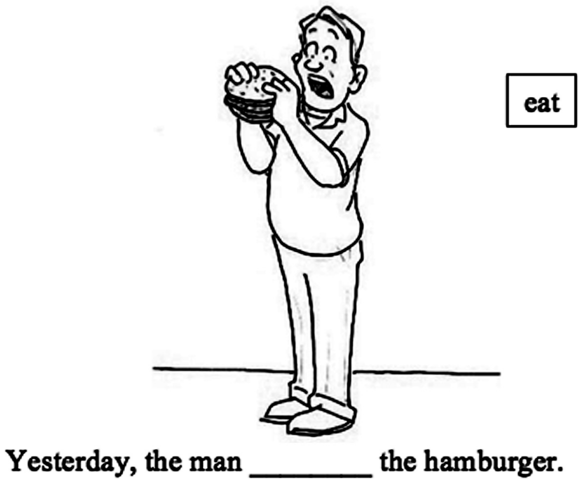

Fig. 4. Sample stimulus for the NAVI test, Experiment 2, to elicit the irregular past tense form, *ate*.

### 3.3. Results

Table 8 presents percent correct responses in each of the nonfinite and finite conditions for the PPA and StrAph participant groups, with these data presented in graphic form in Fig. 4. Analysis of the PPA data using a 2 (group) x 2 (tensed/nontensed) ANOVA revealed a main effect of group ($F$ (1,15) $= 6.68, p = 0.021$), with PPA-G more impaired than PPA-L, and a main effect of tense ($F$ (1,15) $= 30.8$, $p < 0.001$), with tensed verbs more difficult than nontensed ones. There was also an interaction between group and tense ($F$ (1,15) $= 5.28$, $p = 0.036$), with the tense effect greater for the PPA-G group than for the PPA-L group. Post-hoc comparisons showed significantly greater impairment for the PPA-G compared to the PPA-L participants on finite (Mann-Whitney, $Z = -2.01, p = 0.044$), but not on nonfinite (Mann-Whitney, $Z = -1.44, p = 0.149$), verb forms.

Similar analyses of the StrAph data also revealed a significant main effect for group ($F\ (1, 27) = 5.56, p = 0.026$), with StrAg more impaired than StrAn, and a main effect of tense ($F$ (1,27) $= 36.89, p < 0.001$). In addition, as in the PPA group, post-hoc comparisons revealed a significantly greater impairment for finite, but not nonfinite, forms for StrAg compared to the StrAn group (Mann-Whitney, finite: $Z = -2.05, p = 0.04$; nonfinite $Z = -1.37, p = 0.17$).

A 2 (group) x 2 (tensed/nontensed) ANOVA for the two agrammatic groups (PPA-G and StrAg) revealed a main effect of tense ($F$ (1, 25) $= 34.0, p < 0.001$), but no main effect of aphasia type ($F$ (1, 25) $= 1.47, p = 0.237$) or interaction between tense and aphasia type ($F$ (1, 25) $= 0.586, p = 0.451$) (see Fig. 4).

### 3.4. Discussion

As in the previous experiment, these results point to interesting symmetries between StrAph and PPA. In both cases participants with agrammatism scored lower than those with anomic or logopenic impairments on finite verb form production, though not on nonfinite verb form production. In addition, as in Experiment 1, no differences were found between the two agrammatic groups (namely StrAg and PPA-G).

These findings corroborate previous research showing that stroke agrammatic individuals perform sig-

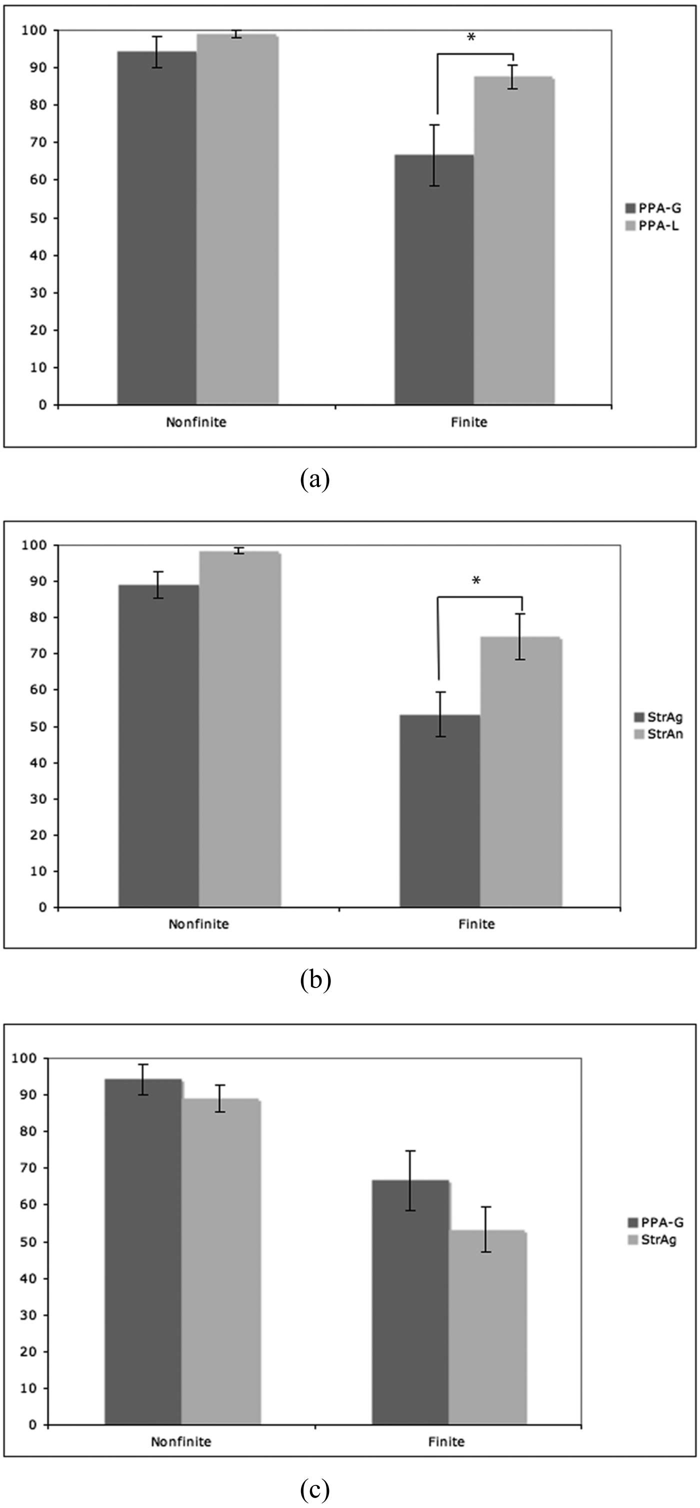

Fig. 4. Percent correct responses (with standard errors), Experiment 2: NAVI nonfinite and finite verb forms for (a) PPA and (b) stroke aphasic participants; (c) displays performance for the two agrammatic groups. $^{*}p < 0.05$, Mann-Whitney test, two-tailed.

nificantly more poorly than individuals with stroke-induced anomic aphasia in the production of inflected verb forms (e.g. [5]). The data also provide important new information distinguishing between agrammatic and logopenic variants of PPA. While individuals with PPA-G show impairments in production of verb tense (mirroring those seen in individuals with agrammatism resulting from stroke in this and previous studies), individuals with PPA-L do not exhibit such deficits.

## 4. Experiment 3. Narrative discourse analysis

Analysis of spontaneous speech enables evaluation of many different aspects of language production, and can reveal deficit patterns indicative of different linguistic impairments. For example, Saffran et al. [50] showed that individuals with stroke-induced nonfluent aphasia exhibit reduced speech rate relative to normal controls in spontaneous speech and that agrammatic individuals in particular use fewer closed-class words, either substitute or omit verb inflections, and produce fewer grammatical sentences compared to healthy control speakers (see also [8,38,49,53,62] and others).

A few studies also have examined narrative production in individuals with PPA, with several noting syntactic and morphosyntactic impairments in nonfluent PPA as compared to normal or fluent PPA speakers [3, 47,55,57,68]. Thompson et al. [55] presented the results of an in depth longitudinal analysis of four patients, then diagnosed with nonfluent PPA, and found low proportions of grammatical sentences and correct verb morphology, high ratios of open- to closed-class words, and high noun to verb ratios in the patients compared to healthy control speakers. Similarly, Gunawardena et al. [33] found fewer complex sentences; Wison et al. [68] found a greater number of syntactic errors; and Ash et al. [2] observed feature omission and incorrect use of grammatical forms in their cohorts of nonfluent PPA speakers. However, the participants with nonfluent PPA studied by Graham et al. [30] showed normal proportions of grammatical morpheme and verb productions in their narrative speech samples, suggesting that the deficit patterns in nonfluent PPA are not in line with agrammatism resulting from stroke.

Part of the lack of complete agreement across studies may relate to the diagnostic classification of nonfluent and fluent PPA, versus more precise classifications of PPA-G, PPA-L, and PPA-S. Notably, only three of the nonfluent PPA patients studied by Thompson et al. [55] showed agrammatic production patterns, which increased in severity over time, whereas one patient (Subject 2) showed a different pattern, characterized by advancing word retrieval difficulty. Notably, based on production patterns, Subject 2 would today be classified as logopenic (and he has since come to autopsy, demonstrating Alzheimer's pathology). In addition, in a more recent study, Thompson et al. [54] found that both PPA-G and PPA-L participants evinced decreased fluency (i.e., nonfluent production), measured by words per minute, compared to healthy control and PPA-S speakers. However, the PPA-G, but not the PPA-L, participants showed reductions in grammatical sentences, correct verb inflection and use of verb argument structure. Thus research examining narrative production in PPA patients is needed, comparing those presenting with PPA-G and PPA-L. Additionally, to our knowledge only two studies have compared the narrative production patterns of PPA and StrAph groups. The Thompson et al. [55] study compared PPA performance to a small group of Broca's aphasic individuals (with agrammatism), with results showing PPA production patterns (for all except Subject 2) very similar to that of the stroke-induced aphasic speakers across a range of linguistic variables. Patterson et al. [47] also examined words per minute (but no other linguistic variables) produced by progressive nonfluent aphasia (PNFA) and nonfluent aphasic (stroke-induced) participant groups. Interestingly, these authors also found no difference between the two groups.

In Experiment 3 we analyzed free narratives obtained from groups of PPA and StrAph speakers. In our analysis, we targeted the following measures: words per minute (WPM), mean length of utterance (MLU), proportion of grammatical sentences, ratio of open- to closed-class words, ratio of nouns to verbs, and proportion of correctly inflected verbs produced. Whereas the first two measures are associated with fluency, the last four are commonly taken to reflect (morpho)syntactic processing, and can be used to detect agrammatic speech.

### 4.1. Method

#### 4.1.1. Participants

Nine PPA-G, 15 PPA-L, 8 StrAg and 15 healthy volunteers recruited from the subject pools of the Aphasia and Neurolinguistics Research Laboratory in Evanston, IL and the Cognitive Neurology and Alzheimer's Disease Center in Chicago, IL, participated in the study.[2] All met the same selection criteria as participants in Experiments 1 and 2. All were right-handed, except three PPA and one StrAg participants. The four groups were matched for education (Kruskal Wallis, $\chi^2(3) = 0.578, p = 0.901$). Aphasic participants ranged in age from 38 to 81 years, with a mean age of 63.0 for PPA-G, 66.5 for PPA-L, and 50.25 for StrAg, and control participants ranged in age from 50 to 74, with a mean of 62.6. Statistical analysis of age across participant groups showed significant differences (Kruskal Wallis, $\chi^2(3) = 12.09, p = 0.007$), with the PPA-G and PPA-L groups significantly older than the StrAg group (Mann-Whitney; PPA-G vs. StrAg: $Z = -2.654, p = 0.006$; PPA-L vs. StrAg: $Z = -2.908, p = 0.004$), although mean ages for the PPA and healthy control groups did not differ (Kruskal Wallis, $\chi^2(2) = 1.337, p = 0.512$) (see Table 1).

The PPA participants showed no evidence of stroke or other neurological disorder and all presented with a history of progressive language deficits with relatively spared performance in other cognitive domains. As in the previous experiments, the MMSE, WMS, Facial Recognition and Trail Making Tests were administered to rule out possible memory, visuoperceptual and attention deficits (see scores in Table 2), with all but three of the participants scoring 24 or higher on the MMSE.

As in Experiments 1 and 2, PPA variants were determined based on scores in the PPVT and the NAT. Both groups showed relatively spared word comprehension, with no significant difference between the groups (PPVT mean: PPA-G = 94.8; PPA-L – 94.0, Mann-Whitney, $Z = -0.331$, $p = 0.741$). On the NAT, the PPA-G group showed impaired syntactic performance, scoring significantly lower than the PPA-L group on noncanonical sentences (NAT noncanonical mean: PPA-G = 30.5; PPA-L = 78.2, Mann-Whitney, $Z = -1.411$, $p = 0.001$), but not on canonical ones (NAT canonical mean: PPA-G = 76.2; PPA-L = 94.5, Mann-Whitney, $Z = -3.377, p = 0.211$).

PPA participants were also administered the WAB, with mean AQs of 79.4 (range: 73.9–85.0) and 88.4 (range: 74.4–94.0) for the PPA-G and PPA-L groups, respectively, which differed significantly (Mann-Whitney, $Z = -3.369, p < 0.001$). WAB fluency scores also differed significantly (Mann-Whitney, $Z = -3.725$, $p < 0.001$) as did performance on the WAB auditory comprehension (Mann-Whitney, $Z = -2.274$, $p = 0.023$) and repetition (Mann-Whitney, $Z = -2.158, p = 0.031$) subtests. However, there was no difference between groups on WAB naming subtest scores (Mann-Whitney, $Z = -0.627, p = 0.558$).

All of the stroke aphasic participants suffered a left-hemisphere stroke, on average 4.6 years prior to the experiment. Participants were classified as agrammatic based on performance on the WAB as well as spontaneous speech production patterns, as explained in Experiment 1. The StrAg participants in this experiment were similar in linguistic performance to those described in the former experiments, with mean WAB-AQ of 75.3 (range: 65.9–87.6), and mean WAB fluency score of 4.4 (range: 4–6) (see Table 3).

There was no significant difference between the two agrammatic groups (i.e. PPA-G and StrAg) on WAB-AQ scores (Mann-Whitney, $Z = 1.734, p = 0.093$).

#### *4.1.2. Procedure*

Narrative language samples were obtained by asking participants to tell the Cinderella story, after looking at a picture book detailing the story. The samples were recorded, transcribed, segmented into utterances, and then coded for linguistic variables using a method described by Thompson et al. [62] (also see [55, 57]). Coding involved several levels of analysis. At the utterance level, coding included identification of sentences versus non-sentential utterances and grammatical versus ungrammatical sentences (e.g. sentences where verbal tense morphology was omitted); at the bound-morpheme level, the use of inflectional morphemes (e.g., verb tense and agreement) was coded; and at the lexical level, all words were coded for open- or closed-class status and for grammatical class (e.g., noun, verb etc.).

### *4.2. Results*

Results on the six outcome measures specified above for each participant group are shown in Table 9. A MANOVA with the six output measures as dependent variables revealed an effect of participant group on all the measures ($p$'s $< 0.001$), except for noun to verb ratio ($p = 0.072$). A post-hoc Bonferroni test was further undertaken to assess between group differences. With regard to measures of fluency, for both WPM and MLU significant differences between the control group and all patient groups were found (p's $< 0.01$ and p's $\leqslant 0.001$, respectively). In addition, the PPA-L group produced significantly more WPM compared

[2] At the time the study was conducted, we were unable to recruit a large enough sample of stroke anomic participants to draw meaningful conclusions.

Table 9
Mean (standard deviation) of outcome measures in narrative analysis, Experiment 3

| Participants | WPM | MLU | % Gram. | % Inflection | N:V ratio | O:C ratio |
|---|---|---|---|---|---|---|
| PPA-G | 57.56 (25.38) | 6.71 (2.08) | 46.56 (18.50) | 81.91 (19.54) | 1.62 (0.84) | 1.06 (0.20) |
| PPA-L | 91.28 (29.61) | 8.28 (1.41) | 80.47 (13.88) | 97.03 (4.85) | 1.11 (0.68) | 0.90 (0.09) |
| StrAg | 52.62 (34.59) | 4.87 (2.02) | 45.13 (21.56) | 82.96 (16.15) | 1.71 (1.51) | 1.54 (0.65) |
| Control | 132.25 (17.43) | 11.92 (3.33) | 92.53 (4.41) | 99.03 (2.06) | 1.19 (0.22) | 0.96 (0.10) |

*Note*: WPM = words per minute, MLU = mean length of utterance, % Gram. = proportion of grammatical sentences, N:V ratio = noun to verb ratio, O:C ratio = open-class to closed-class ratio.

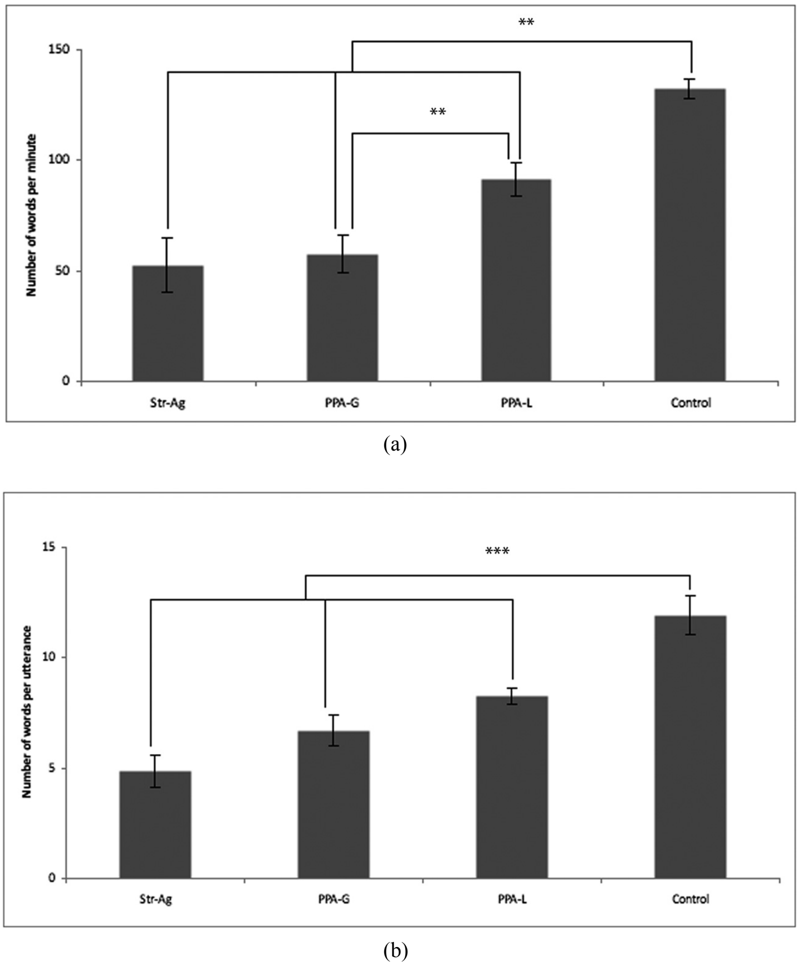

Fig. 5. (a) WPM and (b) MLU by participant group, Experiment 3. $^{***}p < 0.001$; $^{**}p < 0.01$, Mann-Whitney test, two-tailed.

to the PPA-G group ($p = 0.006$), although the two groups did not differ significantly for MLU ($p = 0.827$). No significant differences were noted between the two agrammatic groups for WPM ($p = 1$) or MLU ($p = 0.758$) (see Fig. 5).

Turning next to the proportion of grammatical sentences, no significant difference was found between the control and PPA-L groups ($p = 0.309$). However, both groups differed significantly from the PPA-G group (p's $< 0.001$). The StrAg group likewise displayed a lower proportion of grammatical sentence than controls ($p < 0.001$). In addition, the two agrammatic groups did not differ significantly from each other ($p = 1$). A similar pattern was noted for the proportion of correctly inflected verbs. Although significant differences were found between both agrammatic groups and the control group (PPA-G vs. control, $p = 0.005$, StrAg vs. control, $p = 0.013$), as well as between the PPA-L and PPA-G

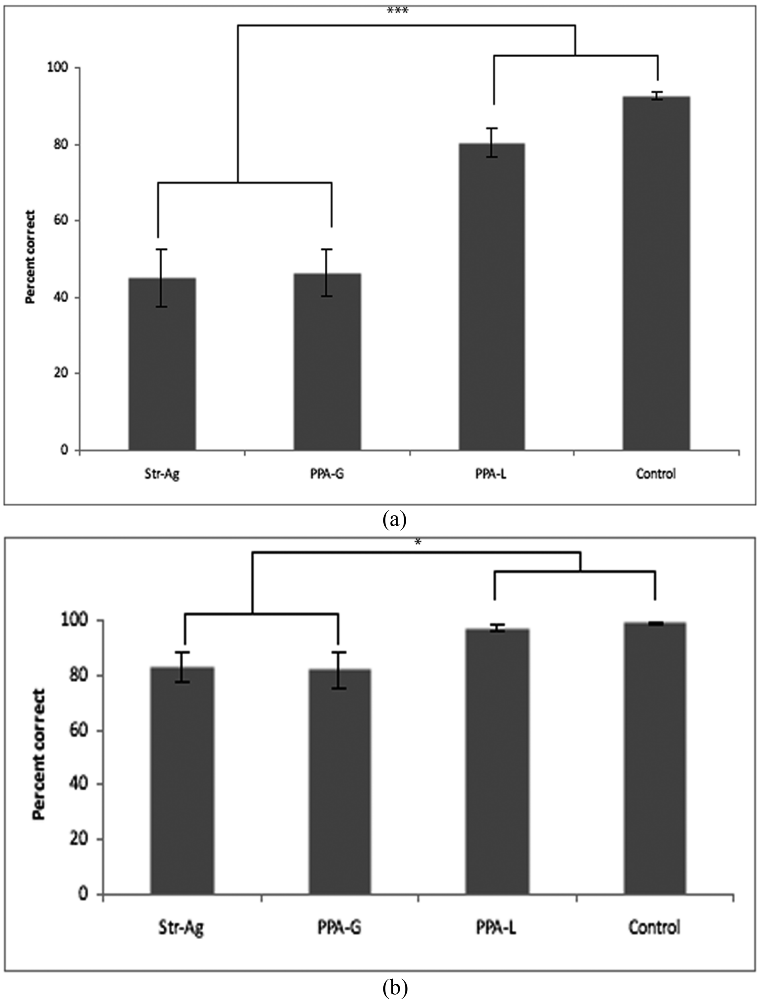

Fig. 6. Proportion of (a) grammatical sentences and (b) correctly inflected verbs by participant group, Experiment 3. $^{*}p < 0.05$, $^{***}p < 0.001$, Mann-Whitney test, two-tailed.

group ($p = 0.018$), the PPA-L and control groups did not differ from one another ($p = 1$). Further, no significant difference was found between the two agrammatic groups ($p = 1$). (see Fig. 6).

For noun-to-verb ratio (N:V ratio), mean ratio for the PPA-G group was higher than that for the PPA-L group, indicating that the agrammatic participants used a higher proportion of nouns in their narratives. However, this difference did not reach significance ($p = 0.245$), due to the large variability within the agrammatic group. As with previous measures, the two agrammatic groups did not differ on N:V ratio ($p = 1$). With regard to open- and closed-class word production, a significant difference was found between the StrAg group and the control group, as well as between the StrAg and PPA-G groups, for open-class to closed-class ratios ($p$'s $< 0.01$). However, PPA-G, PPA-L and control participants did not differ significantly from one another ($p$'s $= 1$). (see Fig. 7).

### 4.3. Discussion

Results of the two fluency measures in the experiment, WPM and MLU, indicate that both PPA groups as well as the stroke-induced agrammatic group were less fluent than healthy controls in their narrative production. The WPM data further show that the agrammatic PPA participants were significantly less fluent than the PPA-L group, in line with previous research (e.g. [2,5, 68]). However, MLU did not differentiate the two patient groups. In support of Thompson et al. [57], these data indicate that use of the term nonfluent to classi-

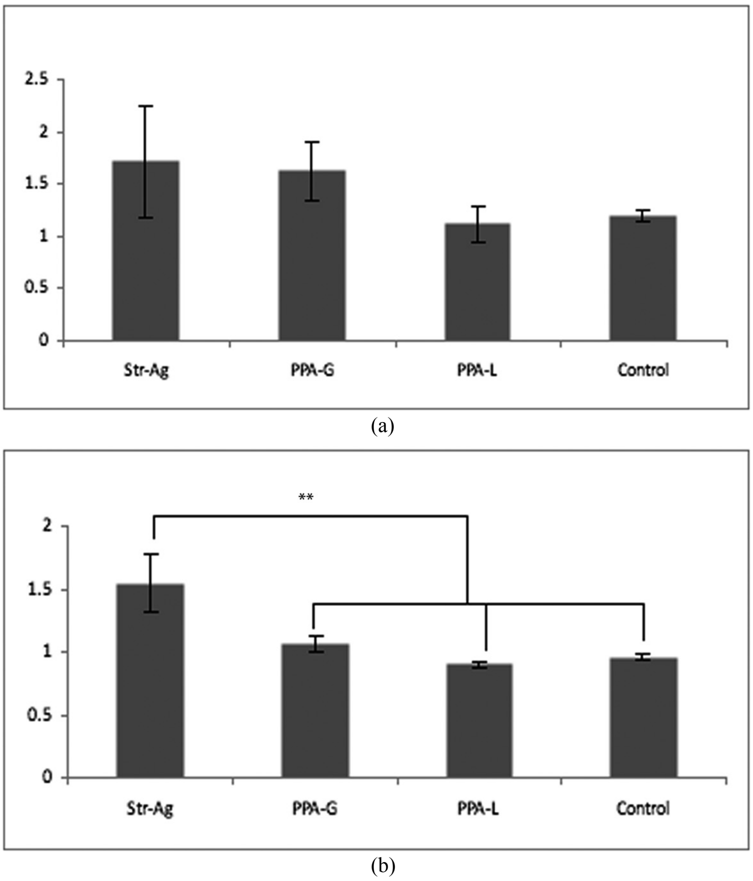

Fig. 7. (a) N:V ratio (b) O:C ratio by participant group, Experiment 3. $^{**}p < 0.01$, Mann-Whitney test, two-tailed.

fy individuals with PPA does not differentiate between agrammatic and logopenic PPA variants. Indeed, both PPA groups demonstrate nonfluent speech compared to normal. However, the two differ in syntactic and morphosyntactic abilities. Importantly, there were no differences in fluency between the two agrammatic groups (i.e. StrAg and PPA-G).

Two of our measures, namely proportion of grammatical sentences and proportion of correctly inflected verbs, clearly indicate that the agrammatic groups are impaired with regard to (morpho)syntactic processing, showing deficits in forming syntactically correct sentences and in using grammatical morphemes to inflect verbs. These findings are consistent with previous ones, suggesting syntactic and morphosyntactic deficits in agrammatic PPA [2,55,57,68]. In contrast, performance of the logopenic PPA participants (i.e., PPA-L) was less impaired than that found in the agrammatic PPA group. These findings suggest relatively unimpaired (morpho)syntactic production ability in this patient group. However, it is worth noting that the proportion of grammatical sentences produced by PPA-L individuals was smaller than that of controls, but this difference did not reach significance (i.e., 80.47% for PPA-L; 92.52% for controls). Thompson et al. [57] and Wilson et al. [68] reported similar patterns, with the PPA-L participants producing significantly fewer grammatical sentences compared to the unimpaired, control speakers, but significantly more grammatical sentences than the PPA-G group. In addition, in the present study, the PPA-L group showed very high levels of correct verb inflection (i.e., a mean of 97%), a pattern also noted in Thompson et al. [57]. Nevertheless, the mild deficit in grammatical sentence production found in PPA-L warrants further investigation to determine the robustness of this effect. For example, grammatical accuracy might be disrupted not because of syntactic processing deficits but rather because of more general word-finding difficulty, which might lead to rephrasing and resultant agrammatic output.

Results of the noun to verb ratio measure again indicate a similar deficit in the two agrammatic groups.

The agrammatic participants (both PPA-G and StrAg) showed a trend towards producing more nouns and fewer verbs, compared to healthy speakers, suggesting a grammatical deficit involving the latter category (the same trend can be seen in the PPA-G group in Thompson et al. [57]). Graham et al. [30], however, did not find evidence of verb production deficits in their nonfluent PPA patients, possibly because their patient cohort included both agrammatic and logopenic variants.

A somewhat surprising result emerged from the open-class to closed-class word production ratio measure. Whereas for StrAg participants, the ratio was higher than that of controls, suggesting, as predicted, a limited use of grammatical, closed-class morphemes, the PPA-G group patterned with the PPA-L and control groups, rather than with the StrAg group, suggesting no impairment for this variable. Notably, Graham et al. [57] likewise did not find a reduced proportion of function words in this group. Based on these results, Graham and colleagues suggested that agrammatism (or progressive nonfluent aphasia) in PPA is not akin to a true agrammatism as seen secondary to stroke aphasia. However, this conclusion is problematic in light of the other agrammatic features displayed by speakers with PPA-G, e.g. reduced proportion of grammatical sentences and correct verb inflection. Notably, Thompson et al. [57] also found significantly reduced argument structure production in their cohort of PPA-G participants, compared to other PPA groups, which also is a pervasive pattern in agrammatism resulting from stroke [5,16,37,42,62]. Indeed, Wilson et al. [68] found a significantly reduced ratio of closed class words in the agrammatic variant of PPA (albeit this was driven by only a few patients). Thompson et al. [55] also found reduced closed class word production in their three agrammatic participants. In addition, this pattern was seen in some of the PPA-G participants in Thompson et al. [57], although significant differences were not found between groups further research comparing open- to closed-class word production in primary progressive and stroke-induced aphasia is needed to determine whether this result reflects a genuine difference between PPA-G and StrAg and, if so, why.

### 4.4. General discussion

In the present study, we examined various measures of syntactic and morphosyntactic performance in several participant groups, including individuals with agrammatic and logopenic variants of primary progressive aphasia (PPA) and those with agrammatic and anomic aphasia resulting from stroke. We examined comprehension and production of canonical and noncanonical sentence structures using the Northwestern Assessment of Verbs and Sentences – Sentence Comprehension Test and Sentence Production Priming Test, and tested production of tensed and nontensed verb forms using the Northwestern Assessment of Verb Inflection. We further examined narrative samples, focusing on measures of speech fluency as well as properties of grammatical speech production. The results showed that agrammatic PPA and stroke-induced aphasic individuals pattern alike on several (morpho)syntactic measures. Although the two groups differed in speech of open- and closed-class words in narrative production, they showed similar deficits in production (and, to a lesser degree, comprehension) of noncanonical sentences, difficulties producing tensed verb forms, and their speech included fewer grammatical sentences and correctly inflected verbs than that of healthy speakers. Our results thus corroborate the findings of Thompson et al. [57] (as well as those of [47,55]), showing that despite the differences between PPA-G and StrAg individuals in disease etiology and mechanism, these two patient groups present with similar language deficit patterns.

In contrast, we found that logopenic PPA speakers did not display the syntactic and morphosyntactic deficits characteristic of the agrammatic groups, and performed significantly better on all tested measures, with the exception of open-to-closed class words in narratives (although other studies show closed-class word production deficits in PPA [55,68]. Notably, syntactic and morphosyntactic performance of the logopenic PPA group was comparable to that of speakers with stroke-induced anomic aphasia in Experiments 1 and 2, and to healthy speakers in Experiment 3. In general, the behavioral pattern of logopenic PPA suggests relatively intact syntactic knowledge, much like that of StrAn speakers.

It can be noted that the poorer performance of the agrammatic groups on pre-experimental language tasks may have predicted these patterns, since participants were categorized as agrammatic based on their ability to produce sentences (i.e., on the Northwestern Anagram Test for PPA participants and in spontaneous speech samples for StrAph). We note, however, that the agrammatic groups in our experiments also were significantly less fluent than the anomic/logopenic groups, based on their WAB fluency scores (the mean fluency score for PPA-G participants across the experiments was 5.52, for StrAg – 4.50, for PPA-L – 8.71, and for StrAn –

7.74). Although fluency and grammatical ability often coincide with one another in StrAg, they do not in PPA-G [57]. We also point out that the NAT only grossly addresses sentence production ability in that this test requires participants to arrange written words to form sentences and, hence, off-line strategies can be used to generate sentences. Further, the spontaneous speech samples used to classify the StrAg participants were derived from the WAB picture description task, which often results in short samples that do not adequately illustrate patient abilities (see Thompson et al. [57], for discussion of the limitations of picture description for evaluating spontaneous speech) and the samples were not thoroughly analyzed. The results for Experiments 1 and 2 were derived from constrained production tasks, and Experiment 1 also examined syntactic comprehension. In addition, Experiment 3, which tested narrative production ability, used a story-telling task and a sophisticated analysis system to quantify linguistic ability.

The systematic comparison of (morpho)syntactic impairments associated with PPA and aphasia resulting from stroke reported in this paper thus revealed symmetry between these neurolinguistic disorders, such that the deficit patterns presented by individuals with the agrammatic variant of PPA parallel, to a large degree, those of agrammatism associated with stroke, whereas the (morpho)syntactic characteristics of the logopenic variant of PPA are similar to those of anomic, stroke-induced, aphasia. These findings indicate that use of linguistically specific and sophisticated tests designed to systematically examine grammatical ability can and should be used to study the language deficit patterns seen in PPA. We further suggest that such tests be used clinically to differentiate between the different subtypes of PPA. This practice will not only contribute to our understanding of PPA and differences in deficits across PPA variants, it also will be informative with regard to how, or if, the language patterns associated with PPA differ from or are similar to those found in stroke aphasia. This latter issue, importantly, may provide insights into how language is processed in the brain. In particular, longitudinal studies examining the neural correlates of progressive (morpho)syntactic deterioration in PPA, compared to static (or improving) neurobehavioral correlates of such processing in stroke-induced aphasia may lead to important discoveries about how the brain computes syntax and morphosyntax.
